# Understanding Cannabis Use After Spinal Cord Injury: A Canadian Survey Study

**DOI:** 10.1016/j.arrct.2025.100498

**Published:** 2025-07-24

**Authors:** David J. Allison, Sanam Ebrahimzadeh, Alexandria Roa Agudelo, Arden Lawson, Daad Kassem, Eldon Loh

**Affiliations:** a Parkwood Institute, Lawson Research Institute, London, Ontario, Canada.; b Department of Physical Medicine and Rehabilitation, Schulich School of Medicine and Dentistry, Western University, London, Ontario, Canada.

**Keywords:** Cannabis, Marijuana, Rehabilitation, Spinal cord injury

## Abstract

**Objective:**

To describe the prevalence and characteristics of cannabis use among Canadians with spinal cord injury (SCI).

**Design:**

Observational study.

**Setting:**

Online survey.

**Participants:**

Canadian adults with any level or severity of SCI (N=80). In total, 136 individuals were screened for participation, of which 80 (61.2% men; mean age: 57.7y) completed the survey.

**Interventions:**

Not applicable.

**Main Outcome Measures:**

Prevalence and frequency of cannabis use; reasons for and against use; perceived effectiveness; adverse events; and modality of cannabis consumption.

**Results:**

Overall, 42.5% of participants reported cannabis use postinjury, and 37.5% were current users, exceeding the general Canadian population’s usage rate (25%). Postinjury, the primary reasons for cannabis use shifted from recreation (25%) to alleviating pain (36.3%) and improving sleep (30%), with users perceiving moderate effectiveness. Reported adverse events were generally mild and infrequent, with fatigue being the most common (11.3%). Edibles replaced smoking as the most common modality of cannabis consumption postinjury. No significant associations were found between cannabis use and demographic variables, including sex, education, or employment status.

**Conclusions:**

This small survey highlights the prevalence and therapeutic motivations for cannabis use among Canadians with SCI, with a shift toward medicinal applications postinjury. With moderate perceived effectiveness and a low prevalence of only mild side effects, future research is warranted to evaluate cannabis’ efficacy and safety in this population and to address barriers to its use, including stigma and health concerns.

The criminalization of cannabis in Canada in 1923^1^ severely limited the study of its potential therapeutic benefits^2^ and contributed to its stigmatization. In July 2001, Health Canada passed legislation approving the use of medicinal cannabis for the treatment of symptoms associated with terminal diseases as well as multiple sclerosis, epilepsy, and side effects related to chemotherapy.^3^ Cannabis has shown promise as a therapeutic for a variety of health conditions, including chronic pain and spasticity.^4^ As such conditions are common after spinal cord injury (SCI) cannabis may provide an effective therapeutic. Furthermore, individuals with SCI have identified a need for additional treatment options for secondary health conditions related to SCI, particularly nonpharmacological options.^5^

Although clinical research in this area remains limited, 2 small randomized controlled trials have explored the use of cannabinoids for neuropathic pain in individuals with SCI. In one study including 42 participants, vaporized cannabis at 2 different Δ9-tetrahydrocannabinol concentrations significantly reduced pain intensity compared with placebo, with the lower dose offering the most favorable risk–benefit profile.^6^ In contrast, a crossover pilot study with 7 participants found no significant difference in pain relief between dronabinol (a synthetic form of Δ9-tetrahydrocannabinol) and an active control (diphenhydramine).^7^ While these preliminary studies offer some insight into the possible role of cannabinoids in managing neuropathic pain in SCI, the limited sample sizes and inconsistent findings underscore the need for more rigorous, large-scale research.

Available data among individuals with SCI in Denmark^8^ and the United States^9^^,^^10^ have shown higher cannabis use rates compared to the general population. In Canada, the legalization of recreational cannabis via the enactment of the Cannabis Act in October 2018^11^ spurred the development and implementation of the Canadian Cannabis Survey as a means of collecting data to better understand how Canadians perceive and use cannabis. This survey has provided critical data related to the general Canadian population; however, data pertaining to cannabis use in the Canadian SCI population is currently unavailable.

The purpose of this study is to describe the prevalence and characteristics of cannabis use among Canadian adults with SCI including usage rates and frequency of cannabis use, reasons for or against cannabis use, perceived effectiveness/adverse events, modality of cannabis consumption, and association with clinical or demographic variables. This study aims to provide preliminary data and increase understanding of the prevalence and characteristics of cannabis use among Canadians with SCI, with the goal of informing future research and treatment.

## Methods

### Study design

This study used an anonymous online questionnaire distributed to adults (aged ≥18y) with any level or severity of SCI from 2 rehabilitation programs. Questionnaire responses were obtained by emailing individuals who had consented to being contacted about future study opportunities or by having a member of the potential participant’s circle of care inform them of the study opportunity. Additionally, the survey was advertised via the “Participate in Research” page on the Spinal Cord Injury Ontario website via a convenience sampling approach. Both consent and the questionnaire were distributed and completed by participants online using the data management software REDCap^a^ (Research Electronic Data Capture) (Vanderbilt University 2004).^11^ The study received ethical approval in October 2020, and the recruitment phase spanned from January 2021 to November 2022.

### Survey

The authors reviewed existing surveys from manuscripts related to cannabis use in individuals with SCI.^9^ From this foundation, an online survey (supplemental appendix S1, available online only at http://www.archives-pmr.org/) was drafted including items pertaining to participant demographics (age, sex, education, employment status), injury characteristics (injury onset, cause, level, severity), as well as items related to cannabis use (frequency, modality, reasons for or against use, effectiveness, and side effects) both pre- and postinjury.

### Participants

Participants included 80 men and women Canadian adults (aged ≥18y) with any level or severity of SCI. To allow for an estimation of the prevalence of cannabis use, participants were allowed to participate regardless of current or prior cannabis use. Participants were recruited from 2 rehabilitation programs. Interested participants were provided with a link to the letter of information. After completing informed consent, participants were able to access and complete the questionnaire anonymously. All participants gave informed consent to experimental procedures, which were approved by the Institutional Review Board and conducted in accordance with the Declaration of Helsinki.

### Statistical analysis

Demographic and injury characteristics were summarized using frequency counts and percentages for nominal variables and means and SDs for continuous variables. Usage rates, frequency of use, modality of cannabis consumption, reasons for or against use, perceived effectiveness, and side effects were described by participants who responded affirmatively to using cannabis before and/or after their injury. Preinjury and postinjury use was cross-tabulated, and the McNemar test was employed to assess whether cannabis use changed from pre- to postinjury. Categorical variables were analyzed using the chi-square test to report comparative data, illustrating findings by demographic data and cannabis use since the injury. Data was analyzed using SPSS 28.^b^ Statistical significance was set at *P*<.05 for all tests.

## Results

### Demographics and clinical characteristics

Of the 136 individuals who were screened for participation and emailed the survey link, 82 agreed to participate; however, 2 were removed because of incomplete surveys, resulting in 80 individuals (58.8%) being included in the analysis. The demographic and injury/clinical characteristics of these participants are provided in table 1. The mean age of participants was 57.7±13.2 years, with an age range of 27-81. Most participants were men (61.2% men vs 38.8% women). The mean number of years since injury onset was 42.8±17.8years (range, 4-77y). Traumatic injuries accounted for 60% of cases. Most of the participants had a postsecondary degree or advanced degree (66.2%) and were retired (43.7%). Cervical (C1-C8) injuries were the most common level of injury (42.5%), while completeness of injury based on the American Spinal Injury Association Impairment Scale score was primarily unknown by participants (80%).

**Table 1:** Demographic and injury characteristics.

Variable	Value
Current age, y, mean (SD) [min-max]	57.7 (13.2) [27-81]
Missing (n)	6
Age at injury, y, mean (SD) [min-max]	42.8 (17.8) [4-77]
Missing	1
Sex	
Male	49 (61.2)
Female	30 (37.5)
Missing	1 (1.3)
Level of education	
Advanced degree	7 (8.7)
Postsecondary degree	46 (57.5)
Secondary school graduation	23 (28.7)
Less than secondary school graduation	3 (3.8)
Missing	1 (1.3)
Employment	
Currently employed – full time	11 (13.8)
Currently employed – part time	8 (10.0)
Not currently employed	26 (32.5)
Retired	35 (43.7)
Missing	0
Level of injury	
Cervical (C1-C8)	34 (42.5)
Thoracic (T1-T12)	24 (30.0)
Lumbar (L1-L5)	16 (20.0)
Unknown	5 (6.2)
Missing	1 (1.3)
Cause of injury	
Motor vehicle accident	24 (30)
Fall	16 (20)
Neurologic/inflammatory condition	16 (20)
Occupational injury	7 (8.8)
Surgical complication	4 (5)
Vascular	4 (5)
Cancer	3 (3.8)
Congenital	3 (3.8)
Other	3 (3.8)

NOTE. Values are n (%) unless otherwise specified.

### Rates and frequency of cannabis use

Rates of cannabis use both pre- and postinjury are shown in table 2. Of the 80 participants surveyed, 41 (51.2%) reported trying cannabis at some point in their lives, 34 (42.5%) reported having used cannabis since their injury, and 30 (37.5%) identified as current users. This is higher than the usage rates of the general Canadian population (25%) reported by the Canadian Cannabis Survey.^12^ While cannabis use was slightly higher postinjury (42.5%) than preinjury (32.5%), a McNemar test showed no significant difference in cannabis use pre- to postinjury (*P*=.14). When considering the frequency of cannabis use among current users, 18 (22.5%) participants reported daily use, 5 (6.3%) reported weekly use, 2 (2.5%) reported monthly use, and 5 (6.3%) reported rarely using cannabis (<1time/mo).

**Table 2:** Rates of cannabis use pre- and postinjury.

Cannabis Use		Postinjury		Total
		No	Yes	
Preinjury	No	39 (48.8)	15 (18.75)	54 (67.5)
	Yes	7 (8.7)	19 (23.75)	26 (32.5)
	Total	46 (57.5)	34 (42.5)	80 (100)

NOTE. Values are n (%).

### Reasons for use, perceived effectiveness, and methods of cannabis use

Data pertaining to reasons for or against cannabis use, perceived effectiveness, adverse events, and modality of cannabis use are presented in table 3. With respect to reasons for using cannabis preinjury, the most common response was for recreation/enjoyment (25.0%), while the most common responses for using cannabis postinjury was reducing pain (36.3 %), followed by improving sleep (30.0%). Among participants who did not use cannabis preinjury, the primary reason was that it was illegal at the time (31.3%). The most frequently reported reasons for not using cannabis after injury were negative health implications (13.8%) and social stigma or fear of judgment (12.5%).

**Table 3:** Reasons for use, perceived effectiveness, and methods of cannabis use.

Item	Preinjury n (%)	Perceived Effectiveness Preinjury Mean (SD)	Postinjury n (%)	Perceived Effectiveness Postinjury Mean (SD)
Reasons for cannabis use				
Recreation/enjoyment	20 (25.0)	46.50 (33.8)	8 (10.0)	71.38 (15.3)
Reducing pain	5 (6.3)	53.80 (26.9)	29 (36.3)	53.10 (27.6)
Reducing spasticity	–	–	16 (20.0)	60.56 (24.4)
Reducing nausea	–	–	4 (5.0)	58.75 (17.5)
Reducing stress/anxiety	5 (6.3)	68.80 (13.6)	12 (15)	70.83 (18.7)
Reducing depression	3 (3.8)	75.00 (10.0)	11 (13.8)	68.00 (21.8)
Improving sleep	5 (6.3)	72.40 (8.08)	24 (30.0)	68.38 (25.6)
Improving appetite	–	–	5 (6.3)	75.75 (17.8)
Reducing the need for other medications	4 (5.0)	89.25 (10.8)	13 (16.3)	69.77 (24.3)
Other	3 (3.8)	50.00 (43.3)	3 (3.8)	85.00 (7.1)
Adverse events associated with cannabis use				
None	15 (18.8)	–	15 (18.8)	–
Fatigue	6 (7.5)	–	9 (11.3)	–
Weight gain	0	–	1 (1.3)	–
Heart palpitations	0	–	0	–
Nausea	1 (1.3)	–	2 (2.5)	–
Low blood pressure	0	–	2 (2.5)	–
Paranoia	2 (2.5)	–	2 (2.5)	–
Reduced motivation	3 (3.8)	–	5 (6.3)	–
Reduced physical capabilities	2 (2.5)	–	4 (5.0)	–
Other	3 (3.8)	–	3 (3.8)	–
Reasons for avoiding cannabis use				
Cost/too expensive	1 (1.3)	–	1 (1.3)	–
Negative health implications	11 (13.8)	–	11 (13.8)	–
Social stigma/fear of judgment	13 (16.3)	–	10 (12.5)	–
Dislike taste/smell	6 (7.5)	–	5 (6.3)	–
Illegal (at the time)	25 (31.3)	–	8 (10.0)	–
Unsure how to obtain cannabis	7 (8.8)	–	4 (5.0)	–
Other	17 (21.3)	–	20 (25.0)	–
Modality of cannabis consumption				
Smoking	22 (27.5)	–	13 (16.3)	–
Vaping	3 (3.8)	–	13 (16.3)	–
Edibles	5 (6.3)	–	21 (26.3)	–
Topical	2 (2.5)	–	5 (6.3)	–
Liquid spray	0	–	7 (8.8)	–
Nabilone	0	–	10 (12.5)	–
Other	3 (3.8)	–	8 (10.0)	–

Participants were asked to rate the effectiveness of cannabis before and after their injury on a scale from 1 to 100 for their intended purpose (table 3). With respect to the most common reasons for cannabis use postinjury, participants perceived cannabis to be moderately effective for their intended purposes, with ratings of 53.1±27.6 for improving pain, and 68.4±25.6 for improving sleep.

In addition to perceived effectiveness, participants were also asked about any adverse events experienced that they associated with cannabis use. The most common response both pre- and postinjury was ‘none’ (18.8%), followed by fatigue (7.5% and 11.3%, respectively). When asked about methods of cannabis consumption, smoking was the most common method preinjury (27.5%), while edibles were the most common method postinjury (26.3%).

### Relationships between demographic characteristics and cannabis use

The distributions of cannabis use postinjury were summarized by sex, employment, and education (table 4). Chi-square analysis was conducted to test the relationship between cannabis use since injury and sex, education, and employment. No significant differences were found for sex (χ^2^=1.88, *df*=2, *P*=.38), education (χ^2^=2.039, *df*=4, *P*=.72), or employment (χ^2^=4.67, *df*=3, *P*=.197).

**Table 4:** Relationships between demographic characteristics and cannabis use.

Variable	Postinjury Cannabis Use, n (%)
**Sex**	
Female	11 (32)
Male	22 (65)
Missing	1 (3)
**Employment status**	
Employed full time	3 (9)
Employed part time	6 (17)
Not employed	10 (30)
Retired	15 (44)
**Education**	
Advanced degree	2 (6)
Postsecondary degree	20 (59)
Secondary school graduation	10 (29)
Less than secondary school graduation	2 (6)
Missing	1 (3)

## Discussion

Despite the critical data generated by the Canadian Cannabis Survey pertaining to the use of cannabis among Canadians, there is a notable gap in our understanding of cannabis use and characteristics among Canadians with SCI. A greater understanding of individuals with SCI is important as it can inform future research and provide insights into the potential therapeutic role of cannabis in managing secondary health complications resulting from SCIs.^11^ This study provides the first data pertaining to usage rates and frequency of cannabis use, reasons for or against cannabis use, perceived effectiveness/adverse events, modality of cannabis consumption, and the association with clinical or demographic variables.

The survey results showed that approximately half of respondents (51.2%) had used cannabis at some point in their life, while 37.5% were current users. Hawley et al^9^ found that only 8% of Americans with SCI reported using cannabis after their injury and were considered current users. In contrast, a more recent US-based survey by Kinnunen et al,^13^ which specifically assessed individuals with SCI and neuropathic pain, reported a much higher current use rate of 76.5%. This elevated rate may reflect the study’s focus on individuals with neuropathic pain, a population more likely to seek alternative pain management strategies. With respect to a Canadian population, a study by Harris et al^14^ found that 38.5% of participants with human immunodeficiency virus were current cannabis users. Further, a survey conducted by Health Canada in 2021 in the general population found that 25% of Canadians reported using cannabis in the past 12 months.^12^ The findings related to cannabis usage from studies by Harris et al^14^ and Health Canada more closely resemble our findings. While our findings align with usage rates reported in Canadians with human immunodeficiency virus and the general population, the substantially higher usage rate (76.5%) reported by Kinnunen et al^13^ among US individuals with SCI and neuropathic pain suggests that both national context (eg, legalization, cultural norms) and specific health conditions may influence cannabis use patterns.

With respect to differences in cannabis use pre- and postinjury, no significant differences were found (preinjury 32.5% vs postinjury 42.5%). In the postinjury phase, there was a marked shift in the reasons for using cannabis. While recreation/enjoyment was the most common reason for cannabis use preinjury (25%), use after injury was primarily related to symptomatic management of medical issues. Pain alleviation (36%) and improving sleep (30%) were the most common reasons for cannabis use postinjury, while only 10% of respondents identified recreation as a reason for using cannabis postinjury. These results align with those from Harris et al,^14^ who found that 65% of current spinal cord-injured American cannabis users used cannabis for medicinal purposes related to their SCI. Similarly, Kinnunen et al^13^ reported that cannabis use among individuals with SCI and neuropathic pain was primarily for symptom management, particularly for reducing pain and improving sleep.

With respect to the modality of cannabis use, participants primarily used cannabis by means of smoking preinjury. Similar results have been shown in the general US and Canadian populations,^12^^,^^15^ and it has been suggested that this relates to users believing that smoking allows for more precise dosing.^16^ Postinjury, our study showed that the preferred modality of cannabis use changed to edibles. This finding corresponds to previous data on Americans with SCI and was suggested to relate to participants seeking to avoid negative respiratory effects associated with smoking.^9^ While the most common response related to adverse effects was ‘none’ (18.8%), the most common affirmative response was fatigue both pre- (7.5%) and postinjury (11.3%).

Demographic factors (sex, level of education, employment status) were not found to be significantly related to cannabis use among our participants. Epidemiologic reports consistently demonstrate that men use cannabis, and seek treatment with cannabis, more frequently than women.^17^ Our study showed a similar trend among Canadians with SCI in that most postinjury cannabis users were men (65%); however, the difference was not significant. With respect to education, data from Health Canada has shown that individuals with a high school diploma or less education had higher rates of cannabis use in the previous 12 months (29%) than those with postgraduate education (17%),^12^ whereas our study found no significant difference between educational attainment and cannabis use. While our sample was highly educated (65% having a postsecondary or advanced degree), our results are corroborated by those of a similar study with a more equal education level distribution (57% high school diploma or less) involving US citizens with SCI, which also showed similar cannabis use rates between education subgroups.^9^

### Study limitations

This study was a cross-sectional study involving a relatively small sample of Canadians with SCI. In addition to the inherent limitations of cross-sectional designs, the small sample may not be generalizable to the entire Canadian SCI population. Furthermore, there is the potential for response bias in that cannabis users may be more motivated to participate. Data for nonresponders were not collected, as these individuals did not provide consent for data use, which limits the ability to assess potential differences between responders and nonresponders. Additionally, the relatively older age of the participants in our sample may influence their experiences and perceptions of cannabis use, potentially limiting the applicability of findings to younger individuals with SCI. While cannabis is legal nationwide in Canada, which may limit geographic differences, and our sample included a balanced numbers of participants who had and had not used cannabis, a future, larger scale study with a wider geographic scope is warranted.

## Conclusions

This study found that approximately half of respondents had used cannabis at some point in their lifetime while 37.5% were current users. The primary reasons for cannabis use postinjury were pain reduction and sleep improvement, with users perceiving moderate effectiveness. For nonusers, the primary reasons for avoiding use included negative health implications, social stigma, and fear of judgment. Reported adverse events were generally mild and infrequent, with fatigue being the most commonly reported. Smoking was a less common modality among Canadians with SCI compared to the general population, with edibles being reported as the preferred method of consumption. Although additional studies are needed to definitively establish the effectiveness of cannabis in SCI, as there are people who will find cannabis use beneficial for their symptoms, clinicians should be prepared to discuss the risks and benefits of cannabis use in those with SCI. These findings provide a preliminary understanding of cannabis use among individuals with SCI in Canada and will help guide further studies and treatment.

## Suppliers

a. REDCap; Vanderbilt University.
b. SPSS, version 28; IBM.

## Disclosure

The investigators have no financial or nonfinancial disclosures to make in relation to this project.

## Appendix Supplementary materials

Image, application 1

## Footnotes

Supplementary material associated with this article can be found, in the online version, at doi:10.1016/j.arrct.2025.100498.
