# Supplementary material for: Understanding Cannabis Use After Spinal Cord Injury: A Canadian Survey Study
Source: Arch Rehabil Res Clin Transl. 2025 Jul 24;7(4):100498. doi: 10.1016/j.arrct.2025.100498 (PMC12750357; doi:10.1016/j.arrct.2025.100498)
Supplement: Supplementary file 1 [file mmc1.docx]

**Supplemental Appendix S1: questionnaire**

Cannabis Use and Perceptions Among Canadians with Spinal Cord Injury


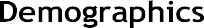


What is your current age?

(years)

Sex Female

Male


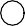

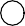

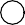

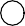


Prefer not to say Other

What was your age at the time of your injury?

*Age of injury cannot be later than current age What was the cause of your injury?

What is the level of your injury? Cervical (C1 - C8) Thoracic (T1 - T12) Lumbar (L1 - L5) Unknown

What is your AIS Score? AIS A (complete)

AIS B AIS C AIS D


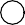

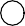

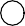

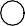

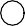

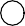

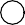

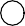

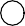


Unknown


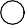

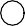

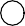

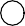


What is your level of education? Less than secondary school graduation Secondary school graduation

Post-secondary degree Advanced degree


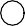

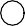

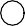

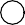


What is your current employment status? Currently employed - Full time Currently employed - Part time Not currently employed Retired

**Pre-injury Cannabis Use**

Did you ever use cannabis prior to your injury? Yes No


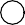

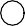


Prior to your injury, how often did you use cannabis? Daily

Weekly Monthly


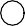

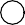

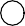

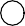


Rarely (less than once per month)

Prior to your injury, for what purpose did you use cannabis?

Recreation / Enjoyment Reducing Pain Reducing Spasticity Reducing Nausea

Reducing Stress / Anxiety Reducing Depression Improving Sleep Improving Appetite

Reducing the need for other medications Other

( select all that apply)

| On a scale from 0-100, how effective do you feel |  | Moderately | Extremely |
| --- | --- | --- | --- |
| cannabis was at providing recreation/enjoyment? | Not Effective | Effective | Effective |

*(Place a mark on the scale above)*

| On a scale from 0-100, how effective do you feel |  | Moderately | Extremely |
| --- | --- | --- | --- |
| cannabis was at reducing pain? | Not Effective | Effective | Effective |

*(Place a mark on the scale above)*

| On a scale from 0-100, how effective do you feel |  | Moderately | Extremely |
| --- | --- | --- | --- |
| cannabis was at reducing spasticity? | Not Effective | Effective | Effective |

*(Place a mark on the scale above)*

| On a scale from 0-100, how effective do you feel |  | Moderately | Extremely |
| --- | --- | --- | --- |
| cannabis was at reducing nausea? | Not Effective | Effective | Effective |

*(Place a mark on the scale above)*

| On a scale from 0-100, how effective do you feel |  | Moderately | Extremely |
| --- | --- | --- | --- |
| cannabis was at reducing stress / anxiety? | Not Effective | Effective | Effective |

*(Place a mark on the scale above)*

| On a scale from 0-100, how effective do you feel |  | Moderately | Extremely |
| --- | --- | --- | --- |
| cannabis was at reducing depression? | Not Effective | Effective | Effective |

*(Place a mark on the scale above)*

| On a scale from 0-100, how effective do you feel |  | Moderately | Extremely |
| --- | --- | --- | --- |
| cannabis was at improving sleep? | Not Effective | Effective | Effective |

*(Place a mark on the scale above)*

| On a scale from 0-100, how effective do you feel |  | Moderately | Extremely |
| --- | --- | --- | --- |
| cannabis was at improving appetite? | Not Effective | Effective | Effective |

*(Place a mark on the scale above)*

| On a scale from 0-100, how effective do you feel cannabis was at reducing the need for other |  | Moderately | Extremely |
| --- | --- | --- | --- |
| medications? | Not Effective | Effective | Effective |
| *(Place a mark on the scale above)* | | | |
| What 'other' purpose did you use cannabis for? |  |  |  |
|  |  |  |  |
| On a scale from 0-100, how effective do you feel cannabis was at improving [preeffectiveness_10]? | Not Effective | Moderately Effective | Extremely Effective |

*(Place a mark on the scale above)*

Do you feel cannabis was more effective than other Yes

medications for your purpose(s)? No


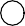

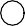


What form(s) of cannabis did you use prior to you injury?

Smoking Vaping Edibles

Topical (applied to skin) Tincture (liquid spray) Nabilone

Other

(select all that apply)

Did you ever experience any negative side effects from cannabis use prior to your injury?

None Fatigue Weight Gain

Heart Palpitations Nausea

Low Blood Pressure Paranoia

Reduced Motivation

Reduced Physical Capabilities Other

(select all that apply)

What was the primary reason(s) you did not use cannabis prior to your injury?

Cost / Too expensive Negative health implications

Social stigma / Fear of judgement Dislike taste / smell

Illegal (at the time)

Unsure how to obtain cannabis Other

(select all that apply)


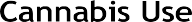


Have you used cannabis since your injury? Yes No


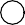

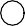

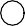

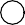

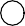

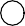

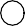


Since your injury, how often do you use cannabis? Daily Weekly Monthly Rarely

I have used cannabis since my injury but no longer use it

Since your injury, for what purpose(s) have you used cannabis?

Recreation / Enjoyment Reducing pain Reducing spasticity Reducing nausea

Reducing stress / anxiety Reducing depression Improving sleep Improving appetite

Reducing need for other medications Other

(select all that apply)

| On a scale from 0-100, how effective do you feel |  | Moderately | Extremely |
| --- | --- | --- | --- |
| cannabis is at improving recreation / enjoyment? | Not Effective | Effective | Effective |

*(Place a mark on the scale above)*

| On a scale from 0-100, how effective do you feel |  | Moderately | Extremely |
| --- | --- | --- | --- |
| cannabis is at reducing pain? | Not Effective | Effective | Effective |

*(Place a mark on the scale above)*

| On a scale from 0-100, how effective do you feel |  | Moderately | Extremely |
| --- | --- | --- | --- |
| cannabis is at reducing spasticity? | Not Effective | Effective | Effective |

*(Place a mark on the scale above)*

| On a scale from 0-100, how effective do you feel |  | Moderately | Extremely |
| --- | --- | --- | --- |
| cannabis is at reducing nausea? | Not Effective | Effective | Effective |

*(Place a mark on the scale above)*

| On a scale from 0-100, how effective do you feel |  | Moderately | Extremely |
| --- | --- | --- | --- |
| cannabis is at reducing stress / anxiety? | Not Effective | Effective | Effective |

*(Place a mark on the scale above)*

| On a scale from 0-100, how effective do you feel |  | Moderately | Extremely |
| --- | --- | --- | --- |
| cannabis is at reducing depression? | Not Effective | Effective | Effective |

*(Place a mark on the scale above)*

| On a scale from 0-100, how effective do you feel |  | Moderately | Extremely |
| --- | --- | --- | --- |
| cannabis is at improving sleep? | Not Effective | Effective | Effective |

*(Place a mark on the scale above)*

| On a scale from 0-100, how effective do you feel |  | Moderately | Extremely |
| --- | --- | --- | --- |
| cannabis is at improving appetite? | Not Effective | Effective | Effective |

*(Place a mark on the scale above)*

| On a scale from 0-100, how effective do you feel cannabis is at reducing the need for other |  | Moderately | Extremely |
| --- | --- | --- | --- |
| medications? | Not Effective | Effective | Effective |
| *(Place a mark on the scale above)* | | | |
| What 'other' purpose did you use cannabis for? |  |  |  |
|  |  |  |  |
| On a scale from 0-100, how effective do you feel cannabis was at improving [posteffectiveness_10]? | Not Effective | Moderately Effective | Extremely Effective |

*(Place a mark on the scale above)*

Do you feel cannabis was more effective than other medications for your purpose(s)?


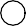
 Yes
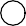
 No

Since your injury, what form(s) of cannabis have you used?

Smoking Vaping Edibles

Topical (applied to skin) Tincture (liquid spray) Nabilone

Other

(select all that apply)

Since your injury have you experienced any negative side effects from cannabis use?

None Fatigue Weight gain

Heart palpitations Nausea

Low blood pressure Paranoia

Reduced motivation

Reduced physical capabilities Other

(select all that apply)

What is the primary reason(s) you have not used cannabis since you injury?

Cost / too expensive Negative health implications

Social stigma / fear of judgment Dislike taste / smell

Illegal (at the time)

Unsure how to obtain cannabis

Other

(select all that apply)
